# New Drugs, Appliances, and Things Medical

**Published:** 1890-09-27

**Authors:** 


### NEW DRUGS, APPLIANCES, AND THINGS MEDICAL.

[All preparations, appliances, novelties, etc., of which a notice is desired, should be sent for The Editor, to care of The Manager, 140, Strand, London, W.C.]

### "BONTHA."

In our notice of this beverage in a recent issue we gave the address of the manufacturers as May-Davis and Co., Westminster Bridge Road. The address of this old-established firm is Esher Street, Westminster, S.W. We are pleased to note that Dr. Norman Kerr, who is a practical authority on wholesome drinks, gives high commendation to Bontha. He states that it possesses the active, fragrant, and enlivening properties of good tea. Bontha contains no tannin, and for that and other reasons well deserves to be tried by all housekeepers.

The *New York Medical Record* gives the following advice from the Pennsylvania Board of Health as regards consumptives: "The duster, and especially that potent distributor of germs—the feather duster, should never be used in the room habitually occupied by a consumptive. The floor, woodwork, and furniture should be wiped with a damp cloth. The patient's clothing should be kept by itself, and thoroughly boiled when washed. It need hardly be said that the room should be ventilated as thoroughly as is consistent with the maintenance of a proper temperature."

